# STORY CIRCLES AS A METHODOLOGY: A PILOT STUDY EXPLORING COGNITIVE PROBLEMS IN AGING

**DOI:** 10.1093/geroni/igz038.2448

**Published:** 2019-11-08

**Authors:** Logan A Sweeder, Nikki Hill, Emily Whitaker, Anushka Tiwari, William Doan

**Affiliations:** The Pennsylvania State University, University Park, Pennsylvania, United States

## Abstract

A Story Circle refers to a group of individuals in a comfortable social environment sharing personal experiences through stories to explore problems shared by a community and facilitate artistic representation of experiences of interest. In this pilot study, we examined the feasibility of Story Circles to facilitate qualitative inquiry of the experience of cognitive problems among older adults. A convenience sample of six cognitively intact, community-dwelling older adults (M=72.5; SD=5.09 years; 83% female) with self-reported cognitive complaints participated in a 90-minute Story Circle as well as a follow-up phone call. Each shared a personal story of experiencing a cognitive complaint and related these experiences to those shared by others in relation to a prompt provided by the group facilitator. Participants reported enjoying the Story Circle experience (M=8.5/10; 10 = extremely positive) and interest in participating in future Story Circles (M=9.3/10; 10 = extremely likely). Common themes included a sense of community established during the group that persisted after its conclusion as well as a normalization of the experience of occasional cognitive problems. Story Circles may be a useful data collection method to enhance understanding of complex phenomena within a social context.

